# Retrospective Cohort Analysis of Survival After SARS-CoV-2 Infection by Vaccination Status in Jamaica, April–December 2021

**DOI:** 10.3390/vaccines13121250

**Published:** 2025-12-17

**Authors:** Karen Webster-Kerr, Andriene Grant, Ardene Harris, Eon Campbell, Deborah Henningham, Marsha Brown, Daidre Rowe, Carol Lord, Romae Thorpe, Tanielle Mullings, Jovan Wiggan, Nicole Martin-Chen, Tonia Dawkins-Beharie, Jacqueline Duncan

**Affiliations:** 1 National Epidemiology Branch, Ministry of Health and Wellness, 15 Knutsford Boulevard, Kingston 5, Jamaica; 2 Family Health Unit, Ministry of Health and Wellness, 10-16 Grenada Way, Knutsford Boulevard, Kingston 5, Jamaica; 3 St. Elizabeth Health Department, Southern Regional Health Authority, 3 Brumalia Road, Mandeville, Jamaica; 4 Department of Community Health and Psychiatry, University of the West Indies, Kingston 7, Jamaica

**Keywords:** COVID-19, SARS-CoV-2, vaccination, survival, COVID-19 death, Kaplan Meier, ChAdOx1 nCoV-19, effectiveness, Jamaica, black population

## Abstract

Background/Objectives: To estimate (a) survival after SARS-CoV-2 infection by COVID-19 vaccination status, and (b) COVID-19 vaccine effectiveness in a middle-income country. Methods: In this retrospective cohort study, secondary analysis of data from the national surveillance and vaccination databases was conducted. The primary outcome was COVID-19 death classified based on the WHO criteria. Data were analysed by vaccination status, age, sex, geographic region, and wave period. Kaplan–Meier curves were plotted; log-rank followed by multiple comparison tests were used to compare survival probabilities. Cox proportional-hazards models with time-varying covariates estimated hazard ratios (HR). Vaccine effectiveness was computed as (1-HR) × 100. Results: A total of 55,299 COVID-19 cases were captured by the national surveillance system between 1 April and 31 December 2021. Of these, 45,774 (1581 vaccinated, 44,193 unvaccinated) were included in the analysis. After a follow-up of 327 days, there were 22 deaths (case fatality rate (CFR) 1.5%) among 1581 COVID-19 vaccinated cases and 1821 deaths (CFR 4.1%) among 44,193 unvaccinated cases. There was one COVID-19 death per 10,000 person days in vaccinated cases compared with 2.7 COVID-19 deaths per 10,000 person days in unvaccinated cases. After adjustment for age, sex, and geographic region, the effectiveness against COVID-19 death across all vaccine types (ChAdOx1 nCoV-19, BNT162b2, Ad26.COV2.S, or BBIBP-CorV) was 68% (95% CI: 51–79). Effectiveness was 75% (95% CI: 59–84) for ChAdOx1 nCoV-19. Vaccine effectiveness across all vaccine types was higher in younger cases, (82% (95% CI: 52–93), 18–64 years vs. 63% (95% CI: 41–77), ≥65 years), females (84% (95% CI: 63–93), females vs. 53% (95% CI: 24–71), males) and those vaccinated in the past 3 months (71% (95% CI: 47–85), past 0–3 months vs. 56% (95% CI: 23–75), 3–6 months). Conclusions: COVID-19 vaccines were effective in preventing COVID-19 death in a population with low vaccination coverage. Limitations of the analysis include the use of surveillance data (under-reporting of cases, missing data), exclusion of partially vaccinated cases, and insufficient data on important confounders (circulating variants and comorbidities).

## 1. Introduction

Coronavirus disease 2019 (COVID-19), caused by SARS-CoV-2, has resulted in over seven million deaths as of June 2025 [1]. The disease presentation may range from asymptomatic cases to severe COVID-19, with respiratory failure and death. Severe outcomes have been linked to older age, male sex, geographic region [2], co-morbidities, and ethnic minorities [3]. Measures to control viral transmission were initially limited to non-pharmaceutical interventions, as there were no effective options for treatment early in the pandemic [4].

As of 26 December 2021, the region of the Americas reported a cumulative total of 101,243,155 confirmed COVID-19 cases, representing 36% of global cases [5]. On 30 March 2021, Jamaica reported higher numbers of confirmed COVID-19 cases per million people (13,565.3) than Barbados (12,854.0) and Trinidad and Tobago (5332.5) [6]. However, by 30 December 2021, after the Omicron variant emerged, Barbados (99,399.8) and Trinidad and Tobago (60,406.9) surpassed Jamaica (32,835.9) [6] in confirmed cases per million people, reflecting a marked shift in the burden of COVID-19 across these countries within nine months.

In December 2020, the first vaccines received emergency use authorization for the prevention of COVID-19 based on their efficacy against symptomatic disease [7]. Since then, meta-analyses have reported vaccine effectiveness against severe COVID-19 (89%), hospitalisation (97%), ICU admission (97%), and death (99%) for persons who are fully vaccinated [8]. However, vaccine effectiveness varies with many factors, such as the type of vaccine, number of vaccine doses, circulating variants, the time since vaccination, and number of booster doses [9,10]. For example, a study evaluating the effectiveness of Pfizer^®^ (BioNTech), Moderna^®^ (mRNA-1273), and AstraZeneca^®^ (ChAdOx1 nCoV-19) vaccines found that pooled vaccine effectiveness (VE) against COVID-19-related mortality was 68% after one dose, rising to 92% after the second dose [11]. In a pooled analysis, Zheng et al. found that vaccine effectiveness was greater for Moderna^®^ (98%), followed by Pfizer^®^ (91%) and CoronaVac^®^ (65%) [8]. Other studies consistently showed the lower efficacy of the AstraZeneca^®^ vaccine compared to Pfizer^®^ (67% vs. 93% in a pooled analysis) [12]. Waning immunity is also documented with vaccine efficacy against SARS-CoV-2 infection and symptomatic COVID-19 decreasing by greater than 20 percentage points at 1 to 6 months after full vaccination [13].

COVID-19 vaccines also provide multifaceted benefits beyond infection prevention [14]. These include economic savings [15,16], improved productivity and social stability during the pandemic, and an accelerated return to normal social and economic life [14]—with many studies quantifying large numbers of deaths averted as a critical outcome of vaccination programmes [15,16].

Despite the benefits of vaccines, low uptake of the COVID-19 vaccine was reported in many low- and middle-income countries (LMICs) due to vaccine hoarding by high-income countries [17] coupled with high levels of vaccine hesitancy [18]. COVID-19 Vaccines Global Access (COVAX) was a global initiative focused on fast-tracking the development and production of COVID-19 vaccines to ensure equitable access worldwide [19]. Jamaica was the first Caribbean country to receive COVID-19 vaccines through the COVAX facility [20], and COVID-19 vaccination began on 10 March 2021, with the AstraZeneca^®^ vaccine [21]. Due to limited supply, priority groups such as essential workers and older persons were initially targeted for vaccination [14]. Subsequently, Pfizer^®^, Johnson & Johnson^®^ (Ad26.COV2.S), Sinopharm^®^ (BBIBP-CorV), and Moderna^®^ (mRNA-1273) were added to the available vaccines, and vaccination of the general population was encouraged [21,22]. However, low vaccine uptake was recorded, with only 26.8% of the population being fully vaccinated by October 2022 [23].

Jamaica’s national surveillance system provided an opportunity to assess vaccine effectiveness in a country with a predominantly black population and low levels of COVID-19 vaccination. This is important as COVID-19 disproportionately affected black populations in high-income countries [24]. Despite the impact of COVID-19 on these populations, lower vaccine uptake and vaccine hesitancy due to inequities, poor access, and distrust of health systems is documented [25,26]. While several COVID-19 vaccine studies from high-income countries were published [27,28], information on vaccine effectiveness in low-resource settings and among black populations or other ethnic minorities is sparse [28,29,30]. In addition, ethnic minorities are generally under-represented in clinical trials, and few reports of vaccine effectiveness in LMICs have emerged. This evidence gap is critical, as studies have shown differences in vaccine efficacy [31] and real-world effectiveness [32] across racial and ethnic groups.

The objectives of this study were to (a) estimate survival of COVID-19 cases in a predominantly black LMIC after full vaccination from 1 April to 31 December 2021 and (b) estimate the effectiveness of COVID-19 vaccines against COVID-19 death.

## 2. Materials and Methods

### 2.1. Study Design

In this retrospective cohort study, secondary analysis of data from the national surveillance and vaccination databases was conducted.

### 2.2. Study Setting

Jamaica has fourteen parishes across four health regions (the South East Regional Health Authority (SERHA), North East Regional Health Authority (NERHA), Western Regional Health Authority (WRHA), and Southern Regional Health Authority (SRHA)). In Jamaica, COVID-19 is a Class 1 Notifiable Disease; hence, all suspected and confirmed cases are reported to the National Surveillance Unit (NSU) within 24 h. A national database captures information on socio-demographic characteristics, comorbidities, and outcomes (e.g., hospitalisation, ICU admission, and death).

COVID-19 vaccination was rolled out by the Ministry of Health and Wellness (MOHW) in March 2021. COVID-19 vaccination sites were administered by the MOHW, and a vaccination database captured data on all persons who were vaccinated. Variables in the vaccination database included socio-demographic data and the type and number of vaccine doses.

### 2.3. Procedure

COVID-19 cases reported to the NSU between 1 April and 31 December 2021 were identified, and relevant sociodemographic and mortality data were extracted from national surveillance databases. Each case was manually matched to the MOHW’s COVID-19 Vaccination Management Platform, a restricted-access registry permitting only sequential, person-level record queries. Two MOHW personnel conducted manual matching by searching NSU case records using either the full first and last names or the first two letters of each name. Potential matches were confirmed by comparing additional identifiers (concatenated sex, age, and residential address), an approach analogous to quality-assured record linkage methods [20].

Individuals for whom no matching COVID-19 Vaccination Management Platform record was found were assumed to have no documented vaccination (“unvaccinated”), whereas cases recorded as vaccinated in the surveillance database but not found in the COVID-19 Vaccination Management Platform were cross-checked with parish health department records. This protocol produced an anonymised, surveillance-based cohort of persons aged ≥ 12 years.

Follow-up for each case began on 10 March 2021 (the date the MOHW vaccination campaign commenced) and continued until death or the fixed censor date of 31 January 2022. Starting follow-up before the infection date allowed us to determine whether and when each individual received vaccine doses before their COVID-19 infection, enabling proper classification of vaccination status at the time of infection [33,34]. Cases were then observed post-infection until death or censoring. This emulates a target-trial cohort design and follows established vaccine effectiveness methodology: defining exposure status at infection and avoiding immortal time bias are critical to obtaining unbiased vaccine effectiveness estimates [34]. In fact, public health guidance highlights that linking case surveillance to immunisation data is essential for determining case vaccination status and monitoring outcomes by vaccination status.

### 2.4. Inclusion and Exclusion Criteria

#### 2.4.1. Inclusion Criteria

The inclusion criteria were as follows: Laboratory-confirmed (RT-PCR/antigen) SARS-CoV-2 infection between 1 April and 31 December 2021, irrespective of clinical signs and symptoms.

#### 2.4.2. Exclusion Criteria

Exclusion criteria included the following: (a) Cases with a date of onset prior to 1 April 2021; (b) cases ineligible for vaccination; (c) imported and repatriated cases; and (d) partially vaccinated, i.e., having had only a single dose of a 2-dose vaccine.

### 2.5. Variable Definitions

A confirmed case was defined as a person with laboratory confirmation of COVID-19 infection, irrespective of clinical signs and symptoms [35].

A COVID-19 death was defined per the WHO International Guidelines for Certification and Classification (Coding) of COVID-19 as Cause of Death, 16 April 2020 [36].

A “vaccinated case” was a COVID-19 case diagnosed at least 14 days after receipt of a second dose of a two-dose vaccine regimen (e.g., ChAdOx1 nCoV-19 or AstraZeneca^®^, and BNT162b2 or Pfizer^®^ vaccine), or at least 14 days after receipt of a one-dose vaccine regimen (Ad26.COV2.S or Johnson & Johnson^®^).

An “unvaccinated case” was a COVID-19 case who did not receive any COVID-19 vaccine or for whom no record was found in the COVID-19 Vaccination Management platform.

Wave periods were identified from Jamaica’s epidemic curves as follows: (a) Wave 2: 1 April–22 June 2021; (b) Wave 3: 23 June–3 December 2021; and (c) Wave 4: 4 December–31 December 2021.

### 2.6. Statistical Analysis

Statistical analyses were performed in Stata 18 (StataCorp, College Station, TX, USA). Kaplan–Meier curves were generated by group and compared by log-rank test (Stata sts test), with manual post hoc pairwise comparisons between subgroups and Bonferroni adjustment. Multivariable Cox regression (stcox) yielded unadjusted (univariable) and adjusted (multivariable) hazard ratios (HRs) and 95% CIs. Vaccination status was treated as a time-varying exposure: individuals were classified as unvaccinated or vaccinated (≥14 days after the second dose, or single-dose regimen). Person-time accrued from cohort entry until COVID-19 death or censoring. Individuals were right-censored at COVID-19 death, death from other causes, or the end of the study period. We adjusted the Cox model for age group and sex, and stratified it by regional health authority to allow each region its own baseline hazard. Vaccine effectiveness (VE) was defined as (1–HR) × 100% and was reported with 95% CIs; absolute measures (risk difference and number needed to vaccinate (NNV)) were also calculated. Incidence rates per 10,000 person-years (with 95% CI) were computed for each subgroup (e.g., sex, age group, health region and vaccine type). We tested the proportional hazards assumption using scaled Schoenfeld residuals (Stata’s estat phtest; *p* = 0.1528), with a non-significant global test indicating no violation. Model discrimination was assessed by Harrell’s concordance C statistics (C = 0.81), indicating good predictive ability. We also examined influence diagnostics (DFBETAs) for each covariate; one extreme outlier was identified, but its removal did not materially change the estimates. To gauge intermediate protection, we repeated the analysis, including the partially vaccinated group (≥14 days after one dose of a two-dose regimen) as a third level of vaccination status. This sensitivity model used the same Cox setup; results were compared to the primary model (with only fully vs. unvaccinated) to check robustness.

### 2.7. Ethical Approval

Ethical review and approval were not sought for this study because the analysis used surveillance data routinely collected as part of Jamaica’s public health response, as mandated by the Public Health (Class 1 Notifiable Diseases) Order, 2003. Personal data were handled in a confidential manner in keeping with the Disaster Risk Management Enforcement Measures of the Government of Jamaica.

## 3. Results

### 3.1. Vaccines Administered and Vaccine Coverage

**Figure 1:**
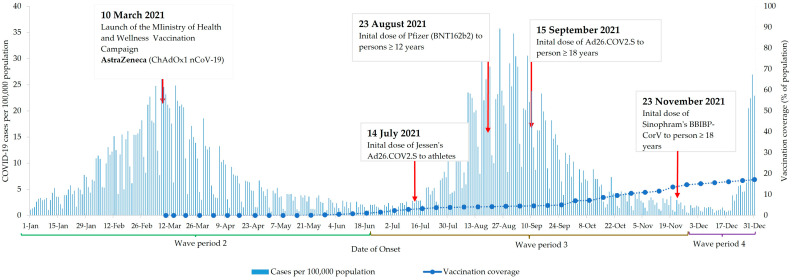
COVID-19 cases and COVID-19 vaccination in Jamaica between 1 January 2021 and 31 December 2021.

The MOHW launched its vaccination campaign on 10 March 2021—close to the peak of the second wave. At the time, AstraZeneca (ChAdOx1 nCoV-19) was the only vaccine available. The Pfizer vaccine was initially administered on 23 August 2021, while Johnson and Johnson (Ad26.COV2.S) and Sinopharm (BBIBP-CorV) vaccines were introduced to the general population on 15 September and 23 November 2021, respectively. As of 31 December 2021, vaccination coverage was 17%. The analysis period ended at the beginning of the fourth wave (Figure 1).

### 3.2. Cohort Selection

**Figure 2:**
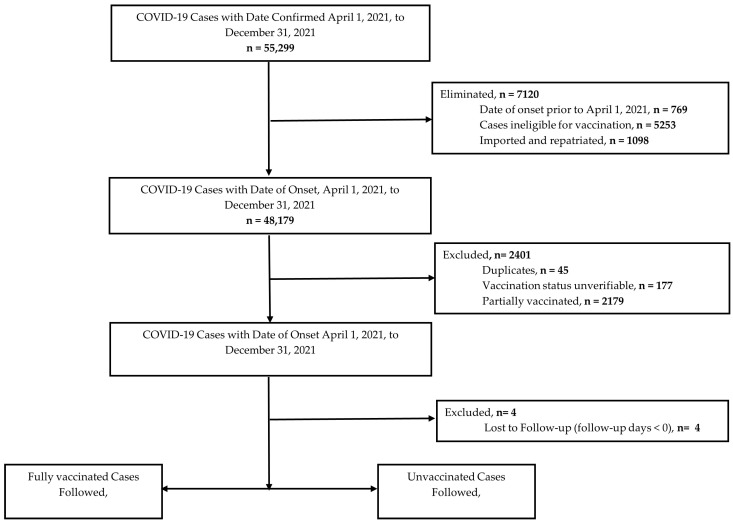
Selection of COVID-19 cases for analysis.

Between 1 April and 31 December 2021, 55,299 COVID-19 cases were captured by the national surveillance system. However, 9521 cases were ineligible for the following reasons: (a) date of onset was prior to study start date (n = 769); (b) ineligible for vaccination (n = 5253); (c) imported/repatriated (n = 1098); (d) partially vaccinated (n = 2179); (e) unverifiable (n = 177); and (f) duplicates (n = 45). The final cohort consisted of 45,774 COVID-19 cases, accounting for four cases that were lost to follow-up (Figure 2).

### 3.3. Demographic Characteristics

Demographic characteristics by COVID-19 mortality and vaccination status are shown in Table 1 and Table 2, respectively. In summary, the median age of COVID-19 cases was 43 years (IQR: 30), with most in the age range 20–39 years (40.0%) and 40–59 years (30.7%). There were more females (58.2%) than males (41.8%), and most cases resided in the south-east health region (46.0%). In general, the majority of deaths were among the elderly (63.3%), with 45.6% occurring in those aged 60–79 years. A similar number of females (50.4%) relative to males (49.6%) died, and the greatest numbers of deaths were observed in the south-east (37.2%) and western (28.6%) health regions (Table 1). Few cases (8.4%) had information recorded on comorbidity status (Table 1).

Of the 45,774 eligible cases, 1581 were vaccinated, and 44,193 were unvaccinated (Table 2). Among vaccinated cases, ChAdOx1 nCoV-19 (AstraZeneca^®^) was the vaccine most commonly used (88.8%), followed by BNT162b2 (Pfizer^®^) (6.4%). Twenty-two deaths were recorded among vaccinated cases, while 1821 deaths were recorded for unvaccinated cases. Of the vaccinated cases, time since vaccination was greater than 6 months in 1124 (71.1%) cases (Table 2). There were significant associations between COVID-19 mortality (*p* < 0.001), age (*p* < 0.001), geographic region (*p* < 0.05), wave period (*p* < 0.001), vaccine type (*p* < 0.001), and vaccination status (Table 1).

### 3.4. COVID-19 Mortality

**Figure 3:**
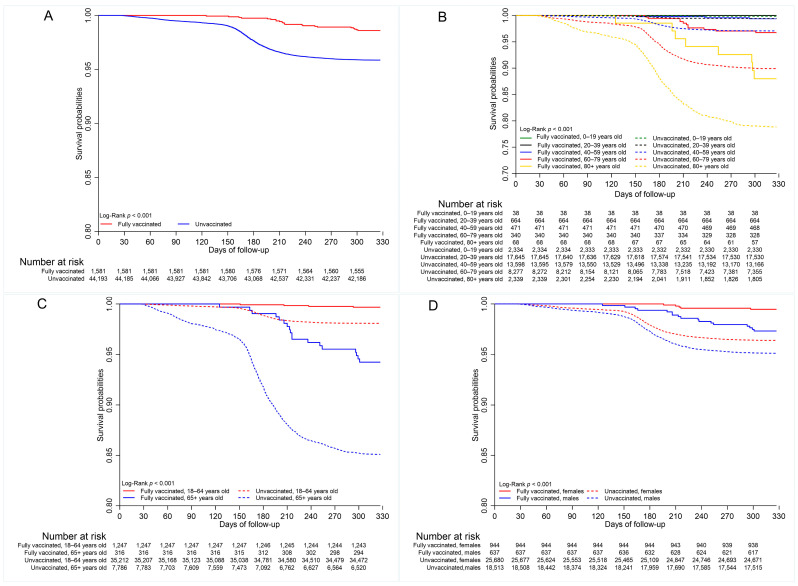

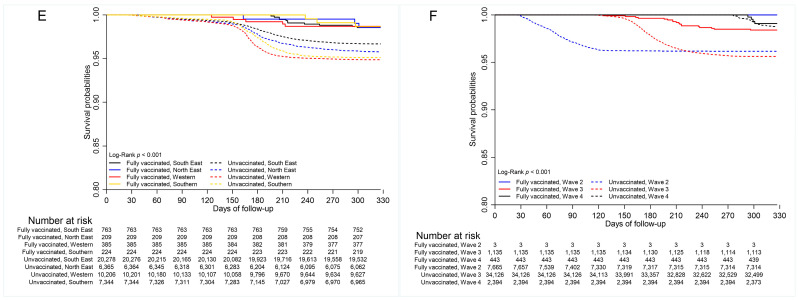
Survival by (**A**) vaccination status (**B**) age group (20-year age category); (**C**) age group (18–64 years vs. 65+ years); (**D**) sex; (**E**) geographic (health) regions; (**F**) wave periods.

There was one COVID-19 death per 10,000 person days in vaccinated cases compared with 2.7 COVID-19 deaths per 10,000 person days in unvaccinated cases over the period under review (Supplemental Table S1). Kaplan–Meier curves are shown in Figure 3. The probabilities of survival were greater for vaccinated (0.988) versus unvaccinated (0.958) cases (*p* < 0.001) (Supplemental Table S2).

Multiple comparison tests indicated differences in mortality between vaccinated and unvaccinated cases in the working-age group (*p* < 0.01) and elderly population (*p* <0.001) (Supplemental Table S3), as well as by sex. The probability of survival was greater for vaccinated individuals in both age categories (Supplemental Table S2, Figure 3C). There was no difference in COVID-19 mortality between unvaccinated females and males who were vaccinated (0.963 vs. 0.979, *p* = 0.999), as well as between the unvaccinated females and unvaccinated males (0.963 vs. 0.950, *p* = 0.120), indicating that males had lower survival than females in the vaccinated and unvaccinated groups (Figure 3D, Supplemental Table S2).

Additionally, multiple comparison tests indicated no difference in survival among geographic regions for vaccinated cases (*p* > 0.05, Supplemental Table S3). Multiple comparison tests further showed that survival was statistically similar for vaccinated cases in all wave periods (*p* > 0.05) (Supplemental Table S3). However, survival probabilities differed significantly between the fully vaccinated and unvaccinated cases for wave periods three (0.989 vs. 0.947, *p* < 0.001) and four (0.986 vs. 0.950, *p* < 0.001). Survival of unvaccinated cases was greater in wave period four than in wave periods two (0.983 vs. 0.964, *p* < 0.001) and three (0.983 vs. 0.955, *p* < 0.001). It was, however, greater in wave period two compared to wave period three (0.964 vs. 0.955, *p* = 0.039).

### 3.5. Vaccine Effectiveness

Vaccine effectiveness against COVID-19 death across all vaccine types was 68% (95% CI: 51–79) after adjusting for age, sex, and geographic region (Table 3). When stratified by age, vaccine effectiveness for persons aged 18–64 years was 82% (95% CI: 52–93). This contrasts with the vaccine effectiveness of 63% in persons aged 65 years and older (95% CI: 41–77). Stratification by sex indicated vaccine effectiveness of 84% (95% CI: 63–93) in females compared with 53% (95% CI: 24–71) in males. Vaccine effectiveness decreased with increasing time since vaccination, declining from 71% (95% CI: 47–85) for persons vaccinated within the past 0–3 months to 56% (95% CI: 23–75) for persons vaccinated in the past 3–6 months. Stratified analysis also indicated vaccine effectiveness varied by health region, with the highest effectiveness occurring in the western region with 78% (95% CI: 46–91) (Table 3).

AstraZeneca (ChAdOx1 nCoV-19) was the most common vaccine administered over the study period. Vaccine effectiveness for ChAdOx1 nCoV-19 (AstraZeneca) was 75% (95% CI: 59–84) after adjustment for age, sex, and geographic region. (Table 3). Similar to the analysis for all vaccine types, vaccine effectiveness for AstraZeneca (ChAdOx1 nCoV-19) against COVID-19 death varied by age, sex, geographic region, and time since vaccination. Vaccine effectiveness was 90% in persons aged 18–64 years (95% CI: 61–98) compared with 69% (95% CI: 48–82) in persons 65 years and older. Persons who were vaccinated in the past 3 months had higher vaccine effectiveness, 87% (95% CI: 65–95), compared with 55% in those vaccinated in the past 3 to 6 months (95% CI: 21–74) (Table 3).

### 3.6. Sensitivity Analyses

Sensitivity analyses were conducted to assess the effect of the inclusion of partially vaccinated cases. When this was performed, vaccine effectiveness across all vaccine types was 76% (95% CI: 60–85) for fully vaccinated cases and 67% (95% CI: 50–78) for partially vaccinated cases (Supplemental Table S4).

## 4. Discussion

This paper adds to the existing literature on real-world experience with COVID-19 vaccines by examining vaccine effectiveness in a Caribbean country with a predominantly black population. Our analysis of national surveillance data suggests that survival probability was greater in vaccinated versus unvaccinated cases. However, survival probability was lower in the vaccinated elderly compared to the vaccinated working-age group. Survival was also lower in males compared to females, irrespective of vaccination status. Among vaccinated cases, there were differences in survival by health region and wave period, suggesting the role of other factors such as structural/health system factors and virologic properties (circulating variants). Nevertheless, there were significantly higher survival probabilities among vaccinated versus unvaccinated individuals in all geographic regions. Vaccine effectiveness across all vaccine types and the main vaccine (ChAdOx1 nCoV-19) were 68% (95% CI: 51–79) and 75% (95% CI: 59–84), respectively, after adjusting for age, sex, and geographic region, with age causing the greatest change in hazard ratio (HR) when compared with the crude model.

Our findings suggest that, in a real-world setting, COVID-19 vaccination remains an essential tool for improving survival in low-resource settings, irrespective of demographic characteristics, and in the black population. The observation of greater survival in vaccinated versus unvaccinated cases is consistent with studies conducted in high-income countries [11,37]. A meta-analysis of seven studies conducted in the United States found that unvaccinated patients were 2.5 times more likely to die from COVID-19 than vaccinated individuals. Fully vaccinated persons were also less likely to develop critical illness, require intensive care, or die from COVID-19 compared to those who were unvaccinated [38].

Other studies examining survival after COVID-19 vaccination reported a 20% lower risk of death among vaccinated compared to unvaccinated patients in Spain [39]. In Peru, research focusing on healthcare workers showed an even more pronounced effect: an 87.5% reduction in mortality risk during the second COVID-19 wave compared to the first, following vaccination in this group [40].

Our study also found disparities in vaccine effectiveness and survival by age and sex. We found that the risk of death was greater, and vaccine effectiveness tended to be lower, in the elderly and among males. These groups have been identified as having a greater risk of severe COVID-19 in other settings [1,2]. Escobar-Agreda et al. (2021) also found higher COVID-19 mortality among male healthcare workers both before and after vaccination, with vaccination producing a more pronounced improvement in survival among males compared to females [40]. In contrast, another survival study reported a slightly lower risk of death among male patients than among females [39]. This study also demonstrated that COVID-19 mortality was higher among older individuals [39].

The significant difference in survival for older COVID-19 cases reinforces the need to prioritise vaccination of this high-risk group. However, the lower vaccine effectiveness reinforces that, in addition to increasing vaccine uptake and combating vaccine hesitancy, other prevention strategies and effective treatment are needed to prevent COVID-19 deaths in older persons and males. This includes access to effective medications, which are shown to reduce the risk of severe COVID-19 in high-risk persons [41,42,43]. Unfortunately, global inequity in vaccines as well as therapeutics has contributed to COVID-19 mortality in these populations in LMIC [44].

Our study also showed significant differences in survival among unvaccinated cases by geographic region, but no regional difference among vaccinated cases, whose survival was greater than the unvaccinated in all geographic regions. This finding suggests a survival advantage for vaccinated cases relative to unvaccinated cases island wide. However, the similar survival rates among vaccinated cases across regions may indicate that vaccination mitigated the effects of regional differences in access to healthcare, COVID-19 management, risk profile of unvaccinated cases, health literacy, or other social determinants of health.

The probability of survival was greatest for both vaccinated and unvaccinated cases during wave period four (4 December 2021 to 31 December 2021), and the gap in survival between these two groups was narrowed for this period. Omicron was the predominant strain circulating in Jamaica during wave 4, and lower mortality is well-documented for Omicron waves in other countries [45]. However, studies show that two-dose mRNA vaccines do not provide adequate protection against symptomatic Omicron infection [46], and Omicron appears to evade natural and acquired immunity [47]. Lower mortality during Omicron waves has been attributed to repeated COVID-19 infections as well as the lower virulence of the Omicron variant compared to the Alpha [48] and Delta variants [49]. Studies suggest that at least three exposures, a combination of vaccine doses and/or COVID-19 infections, are required for protection against Omicron [48]. Although higher vaccine efficacy and effectiveness are reported for the Alpha variant [12], the probability of survival was lowest during wave period two (1 April 2021 to 22 June 2021), when Alpha was the predominant COVID-19 variant in Jamaica.

Vaccine effectiveness against COVID-19 death was 68% (95% CI: 51–79) across all vaccine types and 75% (95% CI: 59–84) for ChAdOx1 nCoV-19. Inclusion of partially vaccinated persons (one dose of the COVID-19 vaccine in a two-dose regimen) in the vaccinated group did not significantly change our findings. Studies examining the effectiveness of a full vaccination schedule from either BNT162b2 (Pfizer), ChAdOx1 (AstraZeneca), Ad26.COV2.S (Janssen), or BBIBP-CorV (Sinopharm) vaccines, found overall effectiveness to be greater than 86% in preventing COVID-19 death [50,51]. However, a 2023 meta-analysis of 20 studies found that efficacy for two doses of AstraZeneca^®^ and Pfizer^®^ were 67% (95% CI, 0.54, 0.80) and 93% (95% CI, 0.85, 1.00), respectively [13] Other studies examining ChAdOx1 have reported effectiveness ranging from 75 to 91% [51,52].

Studies have consistently shown that vaccine-induced immunity wanes over time, with vaccine effectiveness declining notably within 3 to 6 months following vaccination [9,10]. These reductions are consistent with the vaccine effectiveness estimates observed in our study, with almost 20% lower vaccine effectiveness observed for persons vaccinated 3–6 months compared to those vaccinated 0–3 months prior to COVID-19 diagnosis. Although our study included a nine-month follow-up period, it did not fully account for waning immunity, and most persons in our analysis were vaccinated more than 3 months prior to COVID-19 infection. Therefore, the lower vaccine effectiveness observed in our analysis may, in part, reflect the diminished protection associated with time since vaccination. Additionally, variations in vaccine effectiveness may be due to differences in population characteristics, study timeline, circulating variants, and methods used to calculate vaccine effectiveness, as well as the effect of other confounders.

### 4.1. Strengths

Our study fills a gap in the literature on COVID-19 vaccine effectiveness in low- and middle-income countries, many with low vaccination coverage [52]. Other strengths include a long follow-up period and the use of national surveillance and vaccination databases. Robust contact tracing was also conducted during the study period, allowing for the identification of cases. We were also able to account for time to death in COVID-19 cases, which suggests a survival advantage offered by vaccination in our study population. Additionally, the study explored the impact of wave periods on survival between vaccinated and unvaccinated cases, which showed greater differences in survival probabilities by vaccination status when the Alpha and Delta variants were circulating.

### 4.2. Limitations

Limitations include the use of surveillance data, which has inherent drawbacks such as under-reporting, lack of representativeness, and timeliness of reporting [53], as well as missing information. Although the analysis uses data from Jamaica’s national surveillance system, the findings may not be generalisable to the entire Jamaican population due to these limitations. Some important confounders (such as comorbidities) that may influence vaccine receipt and vaccine effectiveness were not adequately captured by the national surveillance system and could not be accounted for in this analysis. Therefore, we were unable to determine the effect of underlying health, reinfections, use of immunosuppressants, and other confounders on vaccine effectiveness. The relatively small number of vaccinated persons also affected the precision of our vaccine effectiveness estimates for sub-groups.

Asymptomatic and vaccinated cases may not have been fully captured in the dataset, and we did not assess effectiveness against symptomatic or any COVID-19 infection. We were also unable to determine effectiveness against other severe outcomes, such as hospitalisation and ICU admission. Few people received mRNA vaccines, so a comparison of different vaccine types was not possible in our analysis.

The full number of COVID-19 cases was not captured in waves two and four due to the start and end dates of the analysis. The start of the vaccination programme was close to the peak of the second wave, while the analysis period ended during the fourth wave. This may have biased study findings due to the inability to capture all cases. The methodology used to determine the points of inflection in this study may have led to possible misclassification of cases and deaths by wave period. This would bias the results to the null if misclassifications were non-differential.

Manual matching of COVID-19 cases with the vaccination database may not have captured all vaccinated cases or may have resulted in linkage errors, leading to misclassification. Non-differential misclassification would bias findings towards the null. Similarly, based on our variable definitions and inclusion criteria, cases vaccinated less than 14 days prior to the analysis would be classified as unvaccinated cases. This would result in an underestimation of vaccine effectiveness since partial vaccination offers some immunity against COVID-19. We were also unable to assess the effectiveness of partial vaccination in the main analysis, since persons who received only one dose of a two-dose vaccine regimen were excluded. However, sensitivity analysis suggests that inclusion of partially vaccinated persons (one dose of the COVID-19 vaccine in a two-dose regimen) in the vaccinated group did not significantly change our findings.

Finally, genomic sequencing was not performed on all positive SARS-CoV-2 samples, limiting the exploration of the influence of variants of concern on vaccine effectiveness. Nevertheless, our results endorse the usefulness of vaccination as an essential tool to reduce the severity of COVID-19 outcomes.

## 5. Conclusions

In conclusion, survival after SARS-CoV-2 infection was greater in vaccinated compared to unvaccinated cases, but this varied by age, sex, and health region. Vaccines were shown to be effective against COVID-19 death in a predominantly black population, however, effectiveness may be lower for elderly persons and men. Waning immunity suggests that booster doses are needed to maintain immunity and reduce COVID-19 deaths.

## Figures and Tables

**Table 1:** Demographic characteristics of COVID-19 cases by mortality status.

Characteristics	Total Cases n = 45,774	COVID-19 Death			
		Yes n = 1843	No n = 43,931	*p*-Value	Case Fatality Rate
	n (%)	n (%)	n (%)		%
Vaccination status					
Vaccinated	1581 (3.5)	22 (1.2)	1559 (3.5)	<0.001	1.5
Unvaccinated	44,193 (96.5)	1821 (98.8)	42,372 (96.5)		4.1
Median age (IQR) ^1^, years	43 (30)	70 (22)	41 (28)	<0.001	
Age group, years					
12–19	2372 (5.2)	3 (0.2)	2369 (5.4)	<0.001	0.1
20–39	18,309 (40.0)	102 (5.6)	18,207 (41.4)		0.6
40–59	14,069 (30.7)	402 (21.8)	13,667 (31.1)		2.9
60–79	8617 (18.8)	840 (45.6)	7777 (17.7)		9.8
≥80	2407 (5.3)	496 (26.9)	1911 (4.4)		20.6
Age group, years					
18–64	36,459 (81.8)	675 (36.7)	35,784 (83.8)	<0.05	1.8
≥65	8102 (18.2)	1167 (63.3)	6935 (16.2)		13.5
Sex					
Female	26,624 (58.2)	929 (50.4)	25,697 (58.5)	0.216	3.5
Male	19,150 (41.8)	914 (49.6)	18,237 (41.5)		4.8
Geographic region					
South East	21,041 (46.0)	684 (37.2)	20,357 (46.3)	<0.050	3.3
North East	6574 (14.4)	271 (14.7)	6303 (14.3)		4.1
Western	10,591 (23.1)	527 (28.6)	10,064 (22.9)		5.0
Southern	7568 (16.5)	361 (19.6)	7207 (16.4)		4.8
Comorbidity					
Yes	3591 (7.8)	1562 (84.8)	2029 (4.6)	<0.001	
No	297 (0.6)	276 (15.0)	21 (0)		
Missing	41,886 (91.5)	5 (0.3)	41,881 (95.3)		
Wave period ^1^					
Wave 2: 1 Apr–22 Jun 2021	7668 (16.8)	293 (14.9)	7375 (16.8)	<0.001	3.6
Wave 3: 23 Jun–3 Dec 2021	35,261 (77.1)	1509 (82.7)	33,752 (76.9)		4.3
Wave 4: 4 Dec–31 Dec 2021	2837 (6.2)	33 (2.5)	2804 (6.5)		1.6
Time Since Vaccination (among vaccinated, n = 1581)					
0–3 months	111 (7.0)	10 (45.5)	101 (6.5)	<0.001	
3–6 months	346 (21.9)	12 (54.5)	334 (21.4)		
>6 months	1124 (71.1)	0 (0)	1124 (72.1)		
Vaccine type (among vaccinated, n = 1581)					
ChAdOx1 nCoV-19	1405 (88.8)	16 (69.6)	1389 (89.0)	<0.001	1.1
BNT162b2	101 (6.4)	4 (17.4)	97 (6.3)		4.0
Ad26.COV2.S	75 (4.7)	2 (8.7)	73 (4.7)		2.7
BBIBP-CorV	1 (0.1)	1 (4.3)	0 (0)		-

^1^ Abbreviation: IQR—interquartile range. Wave periods: Wave 2: 1 Apr–22 Jun 2021; Wave 3: 23 Jun–3 Dec 2021; and Wave 4: 4 Dec–31 Dec 2021.

**Table 2:** Demographic characteristics of COVID-19 cases by vaccination status.

Characteristics	All n = 45,774	Vaccination Status		
		Fully Vaccinated n = 1581	Unvaccinated n = 44,193	*p*-Value
	n (%)	n (%)	n (%)	
COVID-19 death				
Yes	1843 (4.0)	22 (1.4)	1821 (4.1)	<0.001
No	43,931 (96.0)	1559 (98.6)	42,372 (95.9)	
Age group, years				
12–19	2372 (5.2)	38 (2.4)	2334 (5.1)	<0.001
20–39	18,309 (40.0)	664 (42.0)	17,645 (38.5)	
40–59	14,069 (30.7)	471 (29.8)	13,598 (29.7)	
60–79	8617 (18.8)	340 (21.5)	8277 (18.1)	
≥80	2407 (5.3)	68 (4.3)	2339 (5.1)	
Age group, years				
18–64	36,459 (81.8)	1247 (79.8)	35,212 (81.9)	<0.050
≥65	8102 (18.2)	316 (20.2)	7786 (18.1)	
Sex				
Female	26,624 (58.2)	944 (59.7)	25,680 (58.1)	0.205
Male	19,150 (41.8)	637 (40.3)	18,513 (41.9)	
Geographic region				
South East	21,041 (46.0)	763 (48.3)	20,278 (45.9)	<0.05
North East	6574 (14.4)	209 (13.2)	6365 (14.4)	
Western	10,591 (23.1)	385 (24.4)	10,206 (23.1)	
Southern	7568 (16.5)	224 (14.2)	7344 (16.6)	
Comorbidity				
Yes	3591 (7.8)	85 5.4)	3506 (7.9)	<0.001
No	297 (0.6)	2 (0.1)	295 (0.7)	
Missing	41,886 (91.5)	1494 (94.5)	40,392 (91.4)	
Wave period ^1^				
Wave 2: 1 Apr–22 Jun 2021	1405 (88.8)	2 (0.1)	7549 (17.1)	<0.001
Wave 3: 23 Jun–3 Dec 2021	101 (6.4)	1123 (71)	34,186 (77.4)	
Wave 4: 4 Dec–31 Dec 2021	75 (4.7)	457 (28.9)	2461 (5.6)	
Time Since Vaccination (among vaccinated, n = 1581)				
0–3 months	111 (7)	111 (7)	--	__
3–6 months	346 (21.9)	346 (21.9)	--	
>6 months	1124 (71.1)	1124 (71.1)	--	

^1^ Abbreviation: Wave periods: Wave 2: 1 Apr–22 Jun 2021; Wave 3: 23 Jun–3 Dec 2021; and Wave 4: 4 Dec–31 Dec 2021.

**Table 3:** Vaccine effectiveness against COVID-19 deaths in Jamaica.

	Univariable		Multivariable ^1^	
	HR (95% CI)	VE% (95% CI)	HR (95% CI)	VE% (95% CI)
All vaccines	0.34 (0.22–0.52)	66 (48–78)	0.32 (0.21–0.49)	68 (51–79)
Sub-Group				
Sex				
Female	0.15 (0.06–0.36)	85 (64–94)	0.16 (0.07–0.37)	84 (63–93)
Male	0.56 (0.35–0.9)	44 (10–65)	0.47 (0.29–0.76)	53 (24–71)
Age				
18–64	0.18 (0.07–0.47)	82 (53–93)	0.18 (0.07–0.48)	82 (52–93)
≥65	0.37 (0.23–0.59)	63 (41–77)	0.37 (0.23–0.59)	63 (41–77)
Regional Health Authority				
South East	0.44 (0.24–0.8)	56 (20–76)	0.46 (0.26–0.83)	54 (17–74)
North East	0.34 (0.11–1.05)	66 (-5–89)	0.3 (0.1–0.9)	70 (10–90)
Western	0.26 (0.11–0.62)	74 (38–89)	0.22 (0.09–0.54)	78 (46–91)
Southern	0.28 (0.09–0.86)	72 (14–91)	0.26 (0.08–0.79)	74 (21–92)
ChAdOx1 nCoV-19	0.28 (0.17–0.45)	72 (55–83)	0.25 (0.16–0.41)	75 (59–84)
BNT162b2	0.66 (0.17–2.64)	34 (−164–83)	1.46 (0.38–5.67)	−46 (−467–62)
Ad26.COV2.S	0.98 (0.37–2.62)	2 (−162–63)	1.09 (0.43–2.73)	−9 (−173–57)
Time Since Vaccination (among fully vaccinated), months				
0–3	0.29 (0.16–0.54)	0 (1–71)	0.29 (0.15–0.53)	71 (47–85)
3–6	0.5 (0.28–0.88)	0 (1–56)	0.44 (0.25–0.77)	56 (23–75)
>6	--	--	--	--
Vaccine type–ChAdOx1 nCoV-19	0.27 (0.17–0.44)	73 (56–83)	0.25 (0.16–0.41)	75 (59–84)
Subgroup				
Sex				
Female	0.16 (0.07–0.38)	84 (62–93)	0.17 (0.07–0.4)	83 (60–93)
Male	0.41 (0.23–0.74)	59 (26–77)	0.34 (0.19–0.61)	66 (39–81)
Age, years				
18–64	0.1 (0.02–0.39)	90 (61–98)	0.1 (0.02–0.39)	90 (61–98)
≥65	0.31 (0.18–0.52)	69 (48–82)	0.31 (0.18–0.52)	69 (48–82)
Regional Health Authority				
South-East	0.36 (0.18–0.72)	64 (28–82)	0.33 (0.17–0.66)	67 (34–83)
North-East	0.13 (0.02–0.93)	87 (7–98)	0.11 (0.01–0.74)	89 (26–99)
Western	0.22 (0.08–0.59)	78 (41–92)	0.2 (0.07–0.52)	80 (48–93)
Southern	0.29 (0.09–0.89)	71 (11–91)	0.24 (0.08–0.75)	76 (25–92)
Time since vaccination, months				
0–3	0.13 (0.05–0.36)	87 (65–95)	0.13 (0.05–0.35)	87 (65–95)
3–6	0.52 (0.29–0.92)	55 (21–74)	0.45 (0.26–0.79)	55 (21–74)
>6	--	--	--	--

Abbreviation: HR—hazard ratio; VE—vaccine effectiveness. Note. ^1^ Adjusted for age, sex and geographic region; + among those age 18 years and older.

## Data Availability

The original contributions presented in this study are included in the article. The data presented in this study are available on request from the first author due to the data being government data.

## Supplementary Materials

The following supporting information can be downloaded at: https://www.mdpi.com/article/10.3390/vaccines13121250/s1, Table S1: Incidence of COVID-19 Mortality by Demographic Characteristics; Table S2: Kaplan-Meier survival at 327 days, disaggregated by characteristics and vaccination status; Table S3: Multiple Comparison Test Results Following Log Rank Tests; Table S4: Sensitivity Analysis: Vaccine Effectiveness Against COVID-19 Deaths in Jamaica (All Vaccines) Including Partially Vaccinated Cases.
