# Supplementary material for: Retrospective Cohort Analysis of Survival After SARS-CoV-2 Infection by Vaccination Status in Jamaica, April–December 2021
Source: Vaccines (Basel). 2025 Dec 17;13(12):1250. doi: 10.3390/vaccines13121250 (PMC12737689; doi:10.3390/vaccines13121250)
Supplement: Supplementary file 1 [file vaccines-13-01250-s001.zip › vaccines-3897869-supplementary.pdf]

Supplemental Table S1. Incidence of COVID-19 Mortality by Demographic Characteristics

Characteristics	Vaccinated			
	No.	Event	Person time	IR/10,000 person-days
All vaccines	1,581	16	159,688	1.0 (0.6-1.6)
Sex				
Female	944	5	104,741	0.5 (0.2-1.1)
Male	637	17	66,889	2.5 (1.6-4.1)
Age				
12-64	1,247	4	136,955	0.3 (0.1-0.8)
≥65	316	18	34,675	5.2 (3.3-8.2)
Regional Health Authority				
South East	763	11	74,716	1.5 (0.8-2.7)
North East	209	3	21,567	1.4 (0.4-4.3)
Western	385	5	47,109	1.1 (0.4-2.6)
Southern	224	3	28,238	1.1 (0.3-3.3)
Wave period				
Wave 2	3	-	692	
Wave 3	1,135	18	154,916	1.2 (0.7-1.8)
Wave 4	443	4	15,988	2.5 (0.9-6.7)
Comorbidity				
Yes	85	-	163,677	
No	2	-	315	
Missing	1,494	22	7,638	28.8 (19-43.7)
Time since vaccination				
0-3 months	111	10	37,813	2.6 (1.4-4.9)
3-6 months	346	12	112,795	1.1 (0.6-1.9)
>6 months	1,124	-	21,022	
Vaccine type				
ChAdOx1 nCoV-19	1,405	16	159,991	1.0 (0.6-1.6)
BNT162b2	75	2	4,126	4.8 (1.2-19.4)
Ad26.COV2.S	101	4	7,513	5.3 (2-14.2)

Supplemental Table S2. Kaplan-Meier survival at 327 days, disaggregated by characteristics and vaccination status

Characteristics	Vaccinated		Unvaccinated	
	Survival Probability	Error	Survival Probability	Error
**All cases**	0.988	0.0028	0.958	0.001
**Age group, years**				
12-19	1.000	-	0.998	0.0013
20-39	1.000	-	0.994	0.0006
40-59	0.995	0.0034	0.970	0.0015
60-79	0.974	0.009	0.898	0.0034
80+	0.903	0.0376	0.787	0.0086
**Age group, years**				
18-64	0.998	0.0013	0.981	0.0007
65+	0.952	0.0126	0.849	0.0041
**Sex**				
Male	0.979	0.006	0.950	0.0016
Female	0.994	0.0026	0.963	0.0012
**Geographic region**				
South East	0.988	0.0042	0.966	0.0013
North East	0.994	0.0057	0.957	0.0026
Western	0.989	0.0057	0.947	0.0022
Southern	0.986	0.0082	0.950	0.0026
**Wave period**				
Wave Period 2	1.000	-	0.964	0.0022
Wave Period 3	0.988	0.0034	0.955	0.0011
Wave Period 4	0.992	0.0048	0.983	0.0027

Supplemental Table S3. Multiple Comparison Test Results Following Log Rank Tests

	Log-rank test (sts test) p value	Multiple comparisons	chi2(1)	p-value
**Characteristics**		**Fully vaccinated vs. Unvaccinated**	**29.37**	**<0.0001**
Age group (20-year age category)	p < 0.001	Fully vaccinated, 0-19 years old vs. Unvaccinated, 0-19 years old	0.05	1
		Fully vaccinated, 0-19 years old vs. Fully vaccinated, 20-39 years old	0	0
		Fully vaccinated, 0-19 years old vs. Unvaccinated, 20-39 years old	0.22	1
		Fully vaccinated, 0-19 years old vs. Fully vaccinated, 40-59 years old	0.24	1
		Fully vaccinated, 0-19 years old vs. Unvaccinated, 40-59 years old	1.13	1
		Fully vaccinated, 0-19 years old vs. Fully vaccinated, 60-79 years old	1.26	1
		Fully vaccinated, 0-19 years old vs. Unvaccinated, 60-79 years old	4.04	0.444
		Fully vaccinated, 0-19 years old vs. Fully vaccinated, 80+ years old	4.82	0.281
		Fully vaccinated, 0-19 years old vs. Unvaccinated, 80+ years old	9.02	0.027
		Unvaccinated, 0-19 years old vs. Fully vaccinated, 20-39 years old	0.85	1
		Unvaccinated, 0-19 years old vs. Unvaccinated, 20-39 years old	7.96	0.048
		Unvaccinated, 0-19 years old vs. Fully vaccinated, 40-59 years old	4.74	0.294
		Unvaccinated, 0-19 years old vs. Unvaccinated, 40-59 years old	63.21	<0.001
		Unvaccinated, 0-19 years old vs. Fully vaccinated, 60-79 years old	55.85	<0.001
		Unvaccinated, 0-19 years old vs. Unvaccinated, 60-79 years old	241.03	<0.001
		Unvaccinated, 0-19 years old vs. Fully vaccinated, 80+ years old	208.54	<0.001
		Unvaccinated, 0-19 years old vs. Unvaccinated, 80+ years old	543.4	<0.001
		Fully vaccinated, 20-39 years old vs. Unvaccinated, 20-39 years old	543.4	0.497
		Fully vaccinated, 20-39 years old vs. Fully vaccinated, 40-59 years old	4.24	0.395
		Fully vaccinated, 20-39 years old vs. Unvaccinated, 40-59 years old	19.8	1
		Fully vaccinated, 20-39 years old vs. Fully vaccinated, 60-79 years old	21.92	<0.001
		Fully vaccinated, 20-39 years old vs. Unvaccinated, 60-79 years old	70.58	<0.001

	Log-rank test (sts test) p value	Multiple comparisons	chi2(1)	p-value
**Characteristics**		**Fully vaccinated vs. Unvaccinated**	**29.37**	**<0.0001**
		Fully vaccinated, 20-39 years old vs. Fully vaccinated, 80+ years old	84.03	<0.001
		Fully vaccinated, 20-39 years old vs. Unvaccinated, 80+ years old	157.6	<0.001
		Unvaccinated, 20-39 years old vs. Fully vaccinated, 40-59 years old	0.03	1
		Unvaccinated, 20-39 years old vs. Unvaccinated, 40-59 years old	270.91	<0.001
		Unvaccinated, 20-39 years old vs. Fully vaccinated, 60-79 years old	37.8	<0.001
		Unvaccinated, 20-39 years old vs. Unvaccinated, 60-79 years old	1484.96	<0.001
		Unvaccinated, 20-39 years old vs. Fully vaccinated, 80+ years old	139.64	<0.001
		Unvaccinated, 20-39 years old vs. Unvaccinated, 80+ years old	3263.79	<0.001
		Fully vaccinated, 40-59 years old vs. Unvaccinated, 40-59 years old	8.6	0.034
		Fully vaccinated, 40-59 years old vs. Fully vaccinated, 60-79 years old	7.93	0.049
		Fully vaccinated, 40-59 years old vs. Unvaccinated, 60-79 years old	43.99	<0.001
		Fully vaccinated, 40-59 years old vs. Fully vaccinated, 80+ years old	38.82	<0.001
		Fully vaccinated, 40-59 years old vs. Unvaccinated, 80+ years old	105.07	<0.001
		Unvaccinated, 40-59 years old vs. Fully vaccinated, 60-79 years old	0.09	1
		Unvaccinated, 40-59 years old vs. Unvaccinated, 60-79 years old	494.86	<0.001
		Unvaccinated, 40-59 years old vs. Fully vaccinated, 80+ years old	17.95	<0.001
		Unvaccinated, 40-59 years old vs. Unvaccinated, 80+ years old	1325.06	<0.001
		Fully vaccinated, 60-79 years old vs. Unvaccinated, 60-79 years old	16.92	<0.001
		Fully vaccinated, 60-79 years old vs. Fully vaccinated, 80+ years old	9.56	<0.001
		Fully vaccinated, 60-79 years old vs. Unvaccinated, 80+ years old	58.08	<0.001
		Unvaccinated, 60-79 years old vs. Fully vaccinated, 80+ years old	0.13	1
		Unvaccinated, 60-79 years old vs. Unvaccinated, 80+ years old	208.71	<0.001

Characteristics	Log-rank test (sts test) p value	Multiple comparisons	chi2(1)	p-value
		**Fully vaccinated vs. Unvaccinated**	**29.37**	**<0.0001**
		Fully vaccinated, 80+ years old vs. Unvaccinated, 80+ years old	3.7	0.545
Age group (18 - 64 vs 65+ years old)	p < 0.001	Fully vaccinated, 18-64 years old vs. Unvaccinated, 18-64 years old	16.57	<0.001
		Fully vaccinated, 18-64 years old vs. Fully vaccinated, 65+ years old	53.73	<0.001
		Fully vaccinated, 18-64 years old vs. Unvaccinated, 65+ years old	192.39	<0.001
		Unvaccinated, 18-64 years old vs. Fully vaccinated, 65+ years old	23.39	<0.001
		Unvaccinated, 18-64 years old vs. Unvaccinated, 65+ years old	2737.1	<0.001
		Fully vaccinated, 65+ years old vs. Unvaccinated, 65+ years old	20.25	<0.001
Sex	p < 0.001	Fully vaccinated, females vs. Unvaccinated, females	25.22	<0.001
		Fully vaccinated, females vs. Fully vaccinated, males	12.76	0.0024
		Fully vaccinated, females vs. Unvaccinated,males	37.33	<0.001
		Unvaccinated, females vs. Fully vaccinated, males	1.62	1
		Unvaccinated, females vs. Unvaccinated,males	42.87	<0.001
		Fully vaccinated, males vs. Unvaccinated,males	6.54	0.063
Regional Health Authority	p < 0.001	Fully vaccinated, South East vs. Unvaccinated, South East	8.3	0.032
		Fully vaccinated, South East vs. Fully vaccinated, North East	0	1
		Fully vaccinated, South East vs. Unvaccinated, North East	13.98	0.0016
		Fully vaccinated, South East vs. Unvaccinated, South East	0.03	1
		Fully vaccinated, South East vs. Unvaccinated, North East	20.75	<0.001
		Fully vaccinated, South East vs. Fully vaccinated, Southern	0.01	1
		Fully vaccinated, South East vs. Unvaccinated, Southern	18.74	<0.001
		Unvaccinated, South East vs. Fully vaccinated, North East	2.31	1
		Unvaccinated, South East vs. Unvaccinated, North East	11.53	0.0056

	Log-rank test (sts test) p value	Multiple comparisons	chi2(1)	p-value
**Characteristics**		**Fully vaccinated vs. Unvaccinated**	**29.37**	**<0.0001**
		Unvaccinated, South East vs. Unvaccinated, South East	4.81	0.2264
		Unvaccinated, South East vs. Unvaccinated, North East	59.91	<0.001
		Unvaccinated, South East vs. Fully vaccinated, Southern	2.73	0.7904
		Unvaccinated, South East vs. Unvaccinated, Southern	36.51	<0.001
		Fully vaccinated, North East vs. Unvaccinated, North East	3.95	0.3744
		Fully vaccinated, North East vs. Unvaccinated, South East	0.02	1
		Fully vaccinated, North East vs. Unvaccinated, North East	5.76	0.1312
		Fully vaccinated, North East vs. Fully vaccinated, Southern	0.01	1
		Fully vaccinated, North East vs. Unvaccinated, Southern	5.25	0.176
		Unvaccinated, North East vs. Unvaccinated, South East	7.86	0.0408
		Unvaccinated, North East vs. Unvaccinated, North East	7.34	0.0536
		Unvaccinated, North East vs. Fully vaccinated, Southern	4.51	0.2688
		Unvaccinated, North East vs. Unvaccinated, Southern	3.36	0.5352
		Fully vaccinated, Western vs. Unvaccinated, Western	11.28	0.0064
		Fully vaccinated, Western vs. Fully vaccinated, Southern	0	1
		Fully vaccinated, Western vs. Unvaccinated, Southern	10.28	0.0104
		Unvaccinated, Western vs. Fully vaccinated, Southern	6.49	0.0872
		Unvaccinated, Western vs. Unvaccinated, Southern	0.68	1
		Fully vaccinated, Southern vs. Unvaccinated, Southern	5.93	0.1192
Wave period	p < 0.001	Fully vaccinated, Wave 2 vs. Unvaccinated, Wave 2	0.12	0.7319
		Fully vaccinated, Wave 2 vs. Fully vaccinated, Wave 3	0.05	0.8265
		Fully vaccinated, Wave 2 vs. Unvaccinated, Wave 3	0.13	0.714

	Log-rank test (sts test) p value	Multiple comparisons	chi2(1)	p-value
**Characteristics**		**Fully vaccinated vs. Unvaccinated**	**29.37**	**<0.0001**
		Fully vaccinated, Wave 2 vs. Fully vaccinated, Wave 4	0.03	0.8689
		Fully vaccinated, Wave 2 vs. Unvaccinated, Wave 4	0.04	0.8483
		Unvaccinated, Wave 2 vs. Fully vaccinated, Wave 3	14.82	0.0001
		Unvaccinated, Wave 2 vs. Unvaccinated, Wave 3	3.02	0.082
		Unvaccinated, Wave 2 vs. Fully vaccinated, Wave 4	10.12	0.0015
		Unvaccinated, Wave 2 vs. Unvaccinated, Wave 4	40.73	<0.001
		Fully vaccinated, Wave 3 vs. Unvaccinated, Wave 3	20.66	<0.001
		Fully vaccinated, Wave 3 vs. Fully vaccinated, Wave 4	1.11	0.2931
		Fully vaccinated, Wave 3 vs. Unvaccinated, Wave 4	0.88	0.3484
		Unvaccinated, Wave 3 vs. Fully vaccinated, Wave 4	12.6	0.0004
		Unvaccinated, Wave 3 vs. Unvaccinated, Wave 4	55.94	<0.001
		Fully vaccinated, Wave 4 vs. Unvaccinated, Wave 4	0.31	0.5779

Supplemental Table S4. Sensitivity Analysis: Vaccine Effectiveness Against COVID-19 Deaths in Jamaica (All Vaccines) Including Partially Vaccinated Cases

Vaccination Status	No.	Event	Person time, days	IR(95% CI)/10,000 person-days	RD (95% CI)[1]	NNV (95% CI)[1]	Univariable		Multivariable	
							HR (95% CI)	VE (95% CI)	HR (95% CI)	VE (95% CI)
Unvaccinated	42,999	1,821	6,838,541	2.7 (2.5 - 2.7)						
Partially vaccinated	1,668	23	250,188	0.9 (0.6 - 1.3)	0.03 (0.02 - 0.03)	35 (29 - 44)	0.34 (0.22-0.52)	66 (48-78)	0.33 (0.22-0.5)	67 (50-78)
Vaccinated	1,403	16	159,688	1.0 (1.0 - 1.6)	0.03 (0.02 - 0.04)	32 (24 - 51)	0.26 (0.15 - 0.42)	74 (58-84)	0.24 (0.15-0.4)	76 (60-85)

[1] Abbreviation: RD – Risk difference. NNV – Numbers needed to vaccinate
